# Explaining Neural Networks Using Attentive Knowledge Distillation

**DOI:** 10.3390/s21041280

**Published:** 2021-02-11

**Authors:** Hyeonseok Lee, Sungchan Kim

**Affiliations:** 1 Division of Computer Science and Engineering, Jeonbuk National University, Jeollabuk-do 54896, Korea; hslee0390@jbnu.ac.kr; 2 Research Center for Artificial Intelligence Technology, Jeonbuk National University, Jeollabuk-do 54896, Korea

**Keywords:** deep neural networks, visual explanation, attention, knowledge distillation, fine-grained classification

## Abstract

Explaining the prediction of deep neural networks makes the networks more understandable and trusted, leading to their use in various mission critical tasks. Recent progress in the learning capability of networks has primarily been due to the enormous number of model parameters, so that it is usually hard to interpret their operations, as opposed to classical white-box models. For this purpose, generating saliency maps is a popular approach to identify the important input features used for the model prediction. Existing explanation methods typically only use the output of the last convolution layer of the model to generate a saliency map, lacking the information included in intermediate layers. Thus, the corresponding explanations are coarse and result in limited accuracy. Although the accuracy can be improved by iteratively developing a saliency map, this is too time-consuming and is thus impractical. To address these problems, we proposed a novel approach to explain the model prediction by developing an attentive surrogate network using the knowledge distillation. The surrogate network aims to generate a fine-grained saliency map corresponding to the model prediction using meaningful regional information presented over all network layers. Experiments demonstrated that the saliency maps are the result of spatially attentive features learned from the distillation. Thus, they are useful for fine-grained classification tasks. Moreover, the proposed method runs at the rate of 24.3 frames per second, which is much faster than the existing methods by orders of magnitude.

## 1. Introduction

Recent years have witnessed the evolution of deep learning at an astounding rate. For instance, in the image classification task, residual networks [1], the winner of the 2015 ImageNet Large Scale Visual Recognition Challenge (ILSVRC), reduced the error rate from 16.4% in 2012 to 3.6%, outperforming the classification ability of a human. Since then, many enhanced networks in terms of performance and computational demand have been proposed [2,3,4,5]. Due to the superior performance of deep neural networks, they are expected to be deployed to aid in decision making in various real-life problems.

However, generating human-understandable explanations of model predictions is critical for the widespread adoption of deep neural networks. For example, a medical decision support system based on deep neural networks should be trustworthy and able to explain its predictions for patients or clinicians in addition to its ability to accurately diagnose the problem. The excellent performance of deep neural networks is attributed to the learning capability of the models that typically comprise several billions of trainable parameters [1,5,6,7]. Such complexity, however, makes the model’s behavior hard to understand, rendering itself a black box [8].

A large body of approaches has been proposed to create visual explanations of the models’ predictions. They provide visual explanation methods that create saliency maps representing the importance of input features for classification tasks [9,10,11,12,13,14,15,16]. Among them, the approaches in [9,10] were proposed to create visual explanations by measuring the differences in the models’ predictions between normal and occluded inputs. The methods typically sought after the important regions of the input by generating numerous random candidates and aggregating them into a single occlusion mask. Although explanations by these approaches are accurate, a lot of time is required to learn a mask for a given input.

The practical deployment of the explanation methods should satisfy two requirements. First, the methods should run rapidly (i.e., be capable of processing tens of images per second). Second, the method should generate saliency maps of high fidelity that only highlight truly important regions of the image in a fine-grained way [14]. The advantage of the fine-grained explanation is obvious in the case where the small part of an object has a great influence on decision-making. Existing explanation methods do not meet these two requirements. Accurate results of the learning-based approaches come at the cost of large computation time [9,10,14,17]. In contrast, other approaches based on gradient quickly generate the saliency map of the input by a single run of feed-forwarding and back-propagation on the target network. However, their saliency maps are created from the feature maps of the last convolution layers and are thus diffused [11,16].

**Figure 1:**
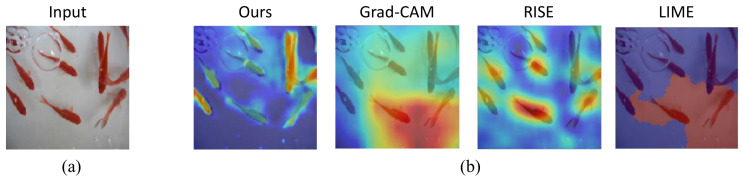
Saliency maps of four explanation methods, the proposed method, Grad-CAM [11], RISE [9], and LIME [10]: (**a**) an input image labeled goldfish, (**b**) explanations that are represented by the pixel-wise multiplication of the input and the saliency maps. The saliency map developed by the proposed method highlights almost all the fishes in the image, whereas the saliency maps of other methods are either blurred or indicate only some of the fishes.

In this paper, we propose a novel approach that generates a saliency map to explain the prediction of the target network by the corresponding surrogate networks (see Figure 1). A saliency map is the most common form of explanation to represent the important input pixels (or features) in a human-understandable manner. The surrogate networks have two network branches: an attentive encoder network that approximates the features of the target network and extracts layer-wise attention, and an explanation network that takes the learned features from the encoder network and generates the final saliency map for the input. We used knowledge distillation (KD) [18] to learn the surrogate networks that explicitly combine the information obtained from the intermediate layers of the encoder network where spatially fined-grained and low level feature activation occurs. As a result, the proposed technique overcomes the previously mentioned limitations of the existing methods and has contributions as follows.

- We propose a knowledge distillation method that transforms the black-box target model into the corresponding surrogate network. The proposed knowledge distillation provides enriched information at various levels to be integrated into a saliency map for the model prediction.
- As a result, the proposed method creates a fine-grained saliency map compared to those of the existing methods. Experiments demonstrate that fusing the multi-level information is beneficial, especially in a fined-grained classification task.
- The proposed method requires no individual learning for the input once the corresponding surrogate networks are trained using the knowledge distillation. Generating a saliency map is done at the inference speed of the surrogate networks, which is significantly faster than the learning-based methods while providing comparable explanations both quantitatively and qualitatively.

## 2. Related Work

In this section, we briefly review recent work on explaining deep neural networks to highlight the advantages of the proposed method.

### 2.1. Learning-by-Perturbation Methods

Methods that belong to this category aim to learn the optimal explanation for a given input by gradually improving a randomly initialized saliency map (i.e., mask) with random perturbations of the input. Perturbing the input image and measuring the corresponding sensitivity of the model prediction is a popular approach to estimate the importance of the input features. Occlusions or masks (as perturbations) are queried to the image repeatedly to determine the optimal saliency map. In particular, this approach measures the prediction difference between the input image and its perturbation assuming that if the important regions of the input are perturbed, the model output (i.e., a confidence score in a classification task) will degrade, compared to that for the original input. These methods learn the feature importance of an input at the pixel-level [14] or at the regional basis [9,17,19].

The method proposed in [9] uses arbitrary input sampling to create a random mask in which each pixel value preserves or perturbs the corresponding part of the input. A method that seeks a locally interpretable model-agnostic explanation was proposed in [10], where an input image is transformed into a group of super pixels and is perturbed by randomly deleting several super pixels.

These methodologies learn to generate a visual explanation by aggregating the model outputs from the perturbations of the input. The resulting saliency maps identify the informative regions representing the object. Furthermore, it is possible to create a fine-grained visual explanation when the perturbation is made at the pixel-level. However, saliency maps generated by those methods are non-deterministic due to the randomized input perturbation. Another disadvantage is that creating a visual explanation in such a way is time-consuming because of numerous inferences for random samples from the input perturbation. For instance, in our experiments, the representative perturbation-based methods (RISE [9] and LIME [10]) processed images in the validation set of ImageNet at the rates of 0.125 and 0.18 fps (frames per second), respectively.

An approach proposed in [17] learns a perturbation mask that captures important regions of the input image as in RISE [9] and LIME [10]. An objective function used in this approach considers finding the smallest part of an image that is sufficient to retain the model output score. Additionally, the need for regularization was also considered to prevent the creation of adversarial evidences when generating a mask.

Wangner et al. [14] used an objective function, similar to [17], to achieve a fine-grained visual explanation by learning pixel-level masks for each of the color channels in the image. Although this method generated fined-grained saliency maps, it requires modification of the nonlinear activation of the original model to avoid generating adversarial saliency maps, whereas the proposed method is non-intrusive.

### 2.2. Activation Map-Based Methods

The activation (i.e., feature) maps of a convolutional neural network (ConvNet) have the regional information. Zeiler and Fergus [15] proposed a method to visualize the role of each layer in a ConvNet using the activation maps of convolutional layers and their counterpart transposed layers. Zhou et al. [16] proposed a technique called class activation mapping (CAM). The method generates a saliency map by linearly combining the weights of fully connected (FC) layers of a ConvNet. Global average pooling (GAP) is applied to the activation maps of the last convolutional layer in the model to calculate the weights of the maps.

### 2.3. Gradient-Based Methods

A gradient represents the amount of change in the output score of the model corresponding to a small change of each dimension of the input. As a result, the gradient can be viewed as a measure of pixel importance to represent how much the pixel contributes to the model prediction. Simonyan et al. [13] proposed a method to extract a class saliency map by accumulating the gradients of the output of the model with respect to the input containing the object class category. The attribution of the class score was evaluated at the pixel-level, and thus resulted in a fined-grained saliency map. Gradient, however, does not directly represent the importance of input features for model prediction. Moreover, the saliency map is often noisy and incorrect compared to the perturbation-based methods.

Selvaraju et al. [11] generalized the CAM by eliminating the need to use GAP and FC in the model. Instead, this method, which is known as Grad-CAM, uses gradients to weigh the activation maps. As both CAM and Grad-CAM use the activation maps of the lowest resolution from the last convolutional layer, their explanations are usually diffused. Although such coarse feature maps are appropriate for general classification or localization tasks, they are not suitable for fine-grained classification tasks wherein each of the classes should be distinguished from the appearance of the target object (even in terms of subtle difference).

## 3. Proposed Method

Section 3.1 formulates the problem by defining objective functions to be solved by the proposed method. Then, Section 3.2 describes the knowledge distillation technique [18] that is used to train surrogate networks in the proposed method and the details of the surrogate networks are given in Section 3.3 and Section 3.4.

### 3.1. Problem Formulation and Overview

For a given image $x_{0}\in R^{d}$ as a *d*-dimensional vector and a target network (T) with parameters $\theta_{T}$, let $Y_{\theta_{T}}\left(x_{0}\right)=\left\{y_{\theta_{T}}^{1}\left(x_{0}\right),y_{\theta_{T}}^{2}\left(x_{0}\right),...,y_{\theta_{T}}^{C}\left(x_{0}\right)\right\}$ be the output of T (i.e., the softmax scores) where $y_{\theta_{T}}^{c}\left(x_{0}\right)\in \left[0,1\right]$ is the score of class *c* and *C* denotes the number of classes. Let $y_{\theta_{T}}^{*}\left(x_{0}\right)\in Y_{\theta_{T}}\left(x_{0}\right)$ be the score of the target class for $x_{0}$ such that $y_{\theta_{T}}^{*}\left(x_{0}\right)=\max y_{\theta_{T}}^{i}\left(x_{0}\right)$ where $y_{\theta_{T}}^{i}\left(x_{0}\right)\in Y_{\theta_{T}}\left(x_{0}\right)$ for given parameters $\theta_{T}$. Note the explanation method just calculates a saliency map corresponding to the predicted class whether or not the prediction is true. Then, the goal of the proposed method is to determine a saliency map $H_{x_{0}}\in {\left[0,1\right]}^{d}$ to explain the model prediction of the target network T, which is given by

(1) $$H_{x_{0}}=\underset{h_{x_{0}}}{\arg\max}y_{\theta_{T}}^{*}\left(h_{x_{0}}⊙x_{0}\right)$$

where ⊙ is an element-wise production. A saliency can be viewed as a 1-channel image with the resolution identical to that of $x_{0}$. We define by explanation ${\hat{x}}_{0}$ the multiplication of $x_{0}$ and its corresponding saliency map $H_{x_{0}}$, ${\hat{x}}_{0}=x_{0}⊙H_{x_{0}}$.

The outstanding prediction capability of deep neural networks is largely due to the hierarchical feature learning through inner layers in the model. This motivates us to combine the operations of intermediate features to draw human-understandable explanations. Unfortunately, the structure of the target network is arbitrary with a huge number of parameters and thus it is often difficult to identify which features should be used to create a meaningful saliency map. We address this concern by considering surrogate networks for the target network that allow the proposed method to explicitly extract the meaningful features for the model prediction.

For this purpose, we use knowledge distillation [18] to implant the knowledge of the target model to the surrogate networks that effectively reveal meaningful information of the target model. Originally, knowledge distillation aims to transfer the prediction capability of the large target network, called the teacher network, to the small network, called the student network, by distilling the concise knowledge representation of the teacher network into the student network.

**Figure 2:**
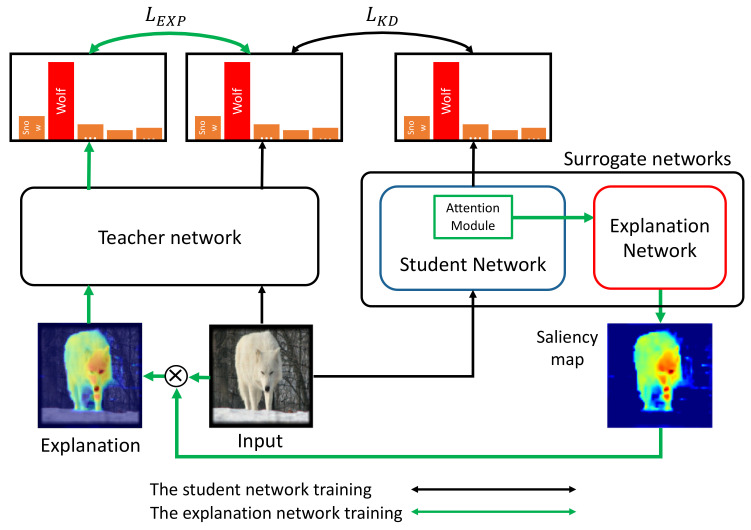
The overall procedure of the proposed explanation method. The black arrows correspond to learning the student network branch, T, of the surrogate networks using the knowledge distillation. The green arrows show the process of training the second branch of the surrogate networks, the explanation network E, to produce a saliency map. $L_{EXP}$ and $L_{KD}$ are loss functions for each training process, which are explained in Section 3.2 and Section 3.4, respectively.

The surrogate networks of the proposed method comprise two network branches to solve Equation (1): an attentive student network (S) with parameters $\theta_{S}$ and an explanation network (E) with parameters $\theta_{E}$ as shown in Figure 2. S encodes the knowledge of T using attention to better learn the features of T. We train S using the knowledge distillation. Another branch E generates a saliency map $H_{x_{0}}$ by exploiting the attentive features transferred from S. The attention modules in S enable S to learn the meaningful intermediate features of T that are expected to contribute to the output score of T, whereas irrelevant or negative features are likely to be suppressed. E takes the information of T that are learned by S as the input and generates the final saliency map. In such a way, the surrogate networks can be viewed as an autoencoder, where T and E are an encoder and a decoder, respectively.

As a result, the proposed method aims to address two sub-problems to achieve the goal specified in Equation (1) as follows: (1) training the student network S to learn the internal behavior of the target network T, so that the explanation network E approximates T in terms of the network output, and (2) explanation ${\hat{x}}_{0}$ should contain meaningful information on the target network prediction for $x_{0}$. In other words, the outputs of T for $x_{0}$ and ${\hat{x}}_{0}$ should be similar. Thus, the first sub-problem can be described as learning S using the knowledge distillation, which is given by

(2) $$\theta_{S}^{*}=\underset{\theta_{E}}{\arg\min}J_{KD}\left(X,\theta_{T},\theta_{S}\right)$$

where $J_{KD}\left(X,\theta_{T},\theta_{S}\right)$ is a cost function for measuring how well the knowledge of T is transferred to S for a given training dataset *X*. In other words, Equation (2) aims to ensure that the output of S is identical to that of T, so that S is an approximate function for T.

The second sub-problem corresponds to the explanation network E. A good explanation method should preserve the important parts of the image by ensuring the corresponding pixels in a saliency map to be close to one, whereas uninformative parts are suppressed, leading to a value of zero. Thus, E aims to generate a faithful explanation for the prediction of T, so that the explanation retrieves the original score of the target class as follows.

(3) $$\theta_{E}^{*}=\underset{\theta_{E}}{\arg\min}J_{EXP}\left(X,\theta_{T},\theta_{E}\right)$$

where $J_{KD}\left(X,\theta_{T},\theta_{S}\right)$ is a cost function that evaluates saliency maps for *X* that were generated by E in terms of retrieving the target class scores from the corresponding explanations.

### 3.2. An Attentive Surrogate Network Learning Using Knowledge Distillation

Training a classifier based on neural networks typically uses only a one-hot-encoded hard label for a given image. However, the image may contain both the information that corresponds to the ground-truth label and that of other recognizable objects in the image. Further, the target object corresponding to the label may have information on other objects with different labels, and thus the model prediction usually results in a soft label. This observation encourages the student network to learn the soft labels instead of the hard ones, which improves the network generalizations.

We applied this technique for leaning the surrogate network in the proposed method. In particular, the target model to be explained is the teacher network to use the knowledge distillation, where the softmax output of the target model can be represented as the soft labels. Unlike the typical setting of the knowledge distillation that uses a small student network, the student network in the proposed method has a number of model parameters similar to T without loosing the prediction capability of T. The proposed method has two advantages related to the use of knowledge distillation. First, any classification network can be the teacher network because the knowledge distillation only requires the output of the last layers of the teacher network. Second, the teacher and the student networks are decoupled, so that a student network can be independently be designed to explaining the model prediction.

Then, the cost function for the knowledge distillation in Equation (2) is written as

(4) $$J_{KD}\left(X,\theta_{T},\theta_{S}\right)=\sum_{x\in X}L_{KD}\left(x\right)=\sum_{x\in X}\alpha L_{soft}\left(x\right)+\left(1-\alpha \right)L_{hard}\left(x\right).$$

where α∈[0,1] is a coefficient to weight two losses $L_{soft}\left(\cdot \right)$ and $L_{hard}\left(\cdot \right)$. For a given image *x*, let $z_{T}^{c}\left(x\right)\in R$ be the pre-softmax output of T for class *c*. Thus we have

(5) $$y_{\theta_{T}}^{c}\left(x\right)=\frac{\exp\left(z_{T}^{c}\left(x\right)\right)}{\sum_{j}\exp\left(z_{T}^{j}\left(x\right)\right)}.$$

Similarly, we define $z_{S}^{c}\left(x\right)\in R$ for S. Then, $L_{soft}\left(\cdot \right)$ is a loss corresponding to soft labels, which is

(6) $$L_{soft}\left(x\right)=t^{2}\cdot D_{KL}\left(\frac{\exp\left(\frac{z_{T}^{c}\left(x\right)}{t}\right)}{\sum_{j}\exp\left(\frac{z_{T}^{j}\left(x\right)}{t}\right)},\frac{\exp\left(\frac{z_{S}^{c}\left(x\right)}{t}\right)}{\sum_{j}\exp\left(\frac{z_{S}^{j}\left(x\right)}{t}\right)}\right)$$

where $D_{KL}\left(\cdot ,\cdot \right)$ is the Kullback–Leibler divergence and *t* is a parameter called temperature. On the other hand, $L_{hard}\left(\cdot \right)$ is a loss corresponding to hard labels as

(7) $$L_{hard}\left(x\right)=CE\left(Y_{\theta_{T}}\left(x\right),Y_{x}\right)$$

where CE(·,·) is a cross entropy and $Y_{x}$ is an one-hot vector to represent the ground-truth hard label of *x*.

### 3.3. Attention-Based Student Network

In this section, we describe the structure of the student network (i.e., the first branch of the surrogate networks) with an emphasis on attention. Attention is a method that aims to obtain information on which part a neural network considers important for the prediction; this originated from a machine translation task in the field of natural language processing [20]. Recently, an approach that only used attention and fully connected layers has achieved the-state-of-the-art performance by outperforming almost all existing natural language processing models [21]. Attention is also popular in vision tasks because it is easy to use and scalable when applying to existing networks [2,4,22,23].

**Figure 3:**
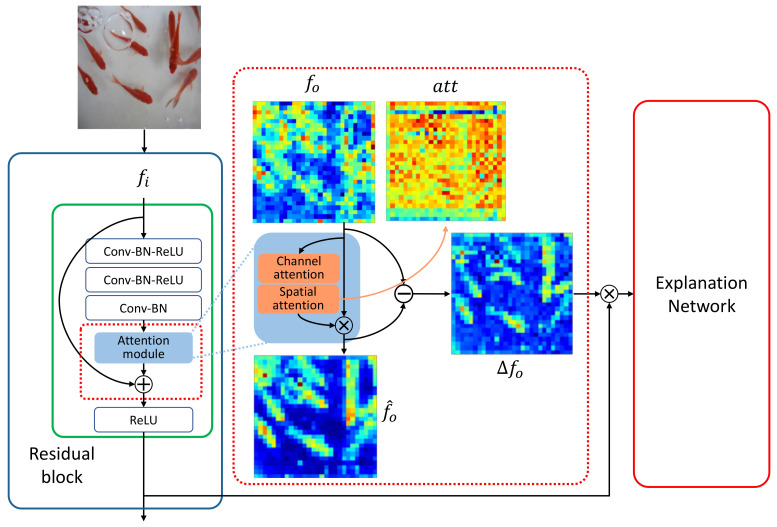
The structure of the attentive student network that consists of multiple residual blocks. *Conv*, *BN*, and *ReLU* stand for a convolution layer, a batch normalization layer, and a rectified linear unit, respectively. An attention module is plugged into a residual block to deliver the attentive features, $▵f_{o}={\hat{f}}_{o}-f_{o}$, for the target network prediction to the explanation network.

The student network is based on ResNet-50 [1] and thus has four residual blocks, the last three of which contain 3–6 attention modules. Each residual block has a pooling layer that halves the dimensions of the last convolutional features of the block, and the number of the feature maps is doubled. Figure 3 shows the attention module in the student network that delivers the attentive features to the explanation network. For an attention module, we used a combination of two branches, channel and spatial attentions, as proposed in [4], which are implemented using lightweight convolutional layers and linear transformations. An attention module can be plugged into an existing network easily to amplify the meaningful regions of the input features to the block.

In particular, a set of features $f_{i}$ is fed into the attention module that is a simple network module with a convolution layer and a fully connected layer for the spatial and channel attention, respectively [4]. Then the features are translated into $f_{o}$, from which we create an attention map $att={\left[0,1\right]}^{dim(f_{0})}$ as illustrated in Figure 3, where $dim(f_{0})$ is the dimension of $f_{o}$. The output of the attention module ${\hat{f}}_{o}$ is then calculated by multiplying the attention map att with the features $f_{o}$. Finally, we take the difference of the features maps, $▵f_{o}$, before and after the attention module (i.e., $▵f_{o}={\hat{f}}_{o}-f_{o}$), which effectively reveals the import part of the features $f_{i}$ and is given as the input of the explanation network.

### 3.4. Explanation Network

**Figure 4:**
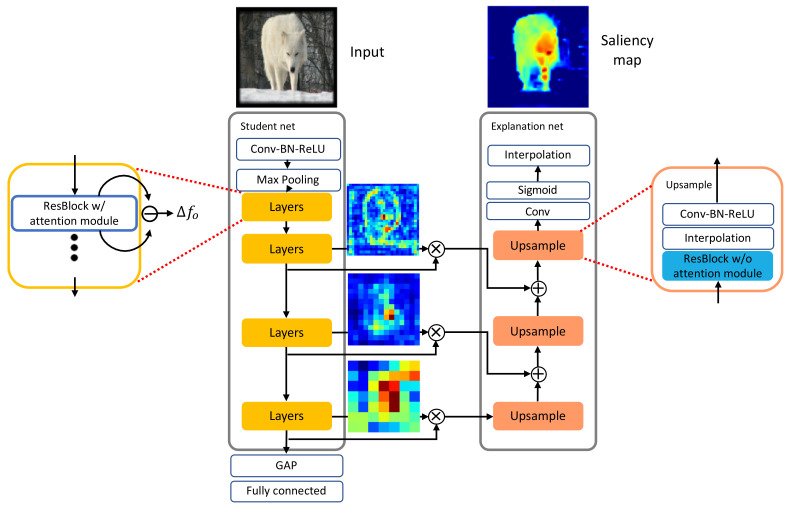
The entire organization of the surrogate networks. The student network creates the multiple features created by the attention modules at the difference scales and transfers them to the explanation network.

As explained earlier, the student network provides the explanation network with information on the attentive features that are taken from the three layers at different scales, as shown in Figure 4. The student and the explanation networks can be viewed as an encoder and a decoder of an autoencoder, respectively. Although the connections between these two networks resemble the skip connections in U-Net [24], their goals are different. In particular, the proposed method delivers the attention features through the connections to identify the influential regions corresponding to the classification. In contrast, U-Net directly concatenates the features maps from the encoder to those of the decoder, with the goal of obtaining better segmentation. The explanation network has three main blocks, called upsample, each of which consists of convolutional and interpolation layers, as shown on the right of Figure 4. In this way, the dimensions of the features in the explanation network grow toward those of the input.

Training the explanation network aims to generate a faithful saliency map for a given input in terms of retrieving the class score on the target network. Then, the cost function for training the explanation network in Equation (3) is given as

(8) $$J_{EXP}\left(X,\theta_{T},\theta_{E}\right)=\sum_{x\in X}L_{EXP}\left(x\right)+\lambda ||H_{x}{\left|\right|}_{1}$$

where λ is a coefficient to weight the $\ell_{1}$-norm of a saliency map $H_{x}$. The $\ell_{1}$ regularization effectively avoids a trivial solution of $H_{x}={\left\{1\right\}}^{d}$, and thus, $\hat{x}=x$. We note that this regularization coincides with previous work that generated perceptually improved images [25,26,27]. As a result, such a benefit also applies to the proposed method.

We formulate $L_{EXP}\left(x\right)$ as in $L_{soft}\left(x\right)$ in Equation (6) by letting t=1, which is given by

(9) $$L_{EXP}\left(x\right)=D_{KL}\left(\frac{\exp\left(z_{T}^{c}\left(x\right)\right)}{\sum_{j}\exp\left(z_{T}^{j}\left(x\right)\right)},\frac{\exp\left(z_{S}^{c}\left(x\right)\right)}{\sum_{j}\exp\left(z_{S}^{j}\left(x\right)\right)}\right)$$

where *c* is the target class of *x*. The proposed method is end-to-end trainable by combining Equations (2) and (3) as follows:

(10) $$\begin{array}{c} \left\{\theta_{S}^{*},\theta_{E}^{*}\right\}=\underset{\theta_{S},\theta_{E}}{\arg\min}J\left(X,\theta_{T},\theta_{S},\theta_{E}\right) \end{array}$$

(11) $$\begin{array}{c} J\left(X,\theta_{T},\theta_{S},\theta_{E}\right)=\sum_{x\in X}L\left(x\right)=\sum_{x\in X}L_{KD}\left(x\right)+L_{EXP}\left(x\right)+\lambda {∥H_{x}∥}_{1} \end{array}$$

where L(·) is an aggregated loss function for the end-to-end training.

## 4. Experiments

We conducted a set of experiments to evaluate the proposed method. The goal of the evaluations was to answer the following questions:

- How do saliency maps generated by the proposed method retrieve the class score that is predicted by the target network for a given input?
- How is the proposed method advantageous over existing explanation methods? In other words, how fast does the proposed method process images? Additionally, are there any downstream tasks that the proposed method performs favorably as compared to the previous methods?

### 4.1. Experimental Setups

**Dataset.** We used four datasets for the experiments: ImageNet [28], CUB-200 [29], Cars [30], and FGVC-Aircraft [31]. ImageNet is a popular large-scale dataset for evaluating generic classification models, whereas CUB200, Cars, and FGVC-Aircraft are datasets that are tailored for fine-grained classification as a downstream task. In particular, CUB-200 is an image dataset that contains 200 bird species that are annotated with bounding box, rough segmentation, and attributes. The Cars dataset contains 196 classes of cars. FGVC-Aircraft is a dataset for the fine grained visual categorization of aircraft by the variant, family, and manufacturer.

Table 1 summarizes the details of these datasets. As images in the test set of ImageNet do not have labels, we used the validation set for evaluating the proposed method. On the other hand, the CUB-200 and Cars datasets have no validation set. Therefore, we used the test set for both validating and testing our model during training as in the case of ImageNet. When training our model using the FGVC-Aircraft dataset, we merged the training and the validation sets into a bigger single training set and used the test set for both validating and testing our model, aiming to achieve better classification performance. As a result, a single entry of each training, validation, and test set is present in Table 1.

**Implementation Details.** We used ResNet-50 that was pretrained on ImageNet as the target network T. We set α in Equation (4) and λ in Equation (8) to 0.5 and $10^{-5}$, respectively. We set the temperature parameter *t* to 1 in Equation (6) for knowledge distillation. The surrogate networks in the proposed method were trained using the Nesterov accelerated stochastic gradient method [32] with a momentum of 0.9. When training the student network S, we set a learning rate to 0.1 for the initial 100 epochs, and then we reduced the learning rate at a scale of 0.1 three times every 30 epochs. The explanation network E was trained as in the case of S but with the different intervals to adjust the learning rate, which were 10 epochs for the initial training and 2 epochs of the duration to reduce the learning rate, respectively. When using the datasets for the fine-grained classifications, we fine-tuned T by using the SGD optimizer with a momentum of 0.9 and an initial learning rate of 0.01. We trained the model for 100 epochs with the learning rate halved every 20 epochs.

We used PyTorch 3.6 [33] to implement the proposed method, and trained the networks using an NVIDIA Titan XP GPU. Table 2 shows the training results of the student network in terms of the top-1 accuracy on each of the datasets. The top-1 accuracy, $acc(\theta_{\psi },X)$, of model ψ with parameters $\theta_{\psi }$ on the test dataset *X* is given as follows.

(12) $$acc\left(\theta_{\psi },X\right)=\frac{\sum_{x\in X}I\left(c_{pred,x}=c_{T,x}\right)}{\left|X\right|}$$

where $c_{T,x}$ is the true class of *x* and $c_{pred,x}={\arg\max}_{i}y_{\theta_{\psi }}^{i}\left(x\right)$ for the given softmax scores of x∈X, $Y_{\theta_{\psi }}\left(x\right)=\left\{y_{\theta_{\psi }}^{i}\left(x\right)\right\}$.

### 4.2. Quantitative Evaluations

#### 4.2.1. Evaluation Methods

**Figure 5:**
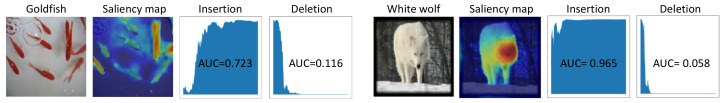
Two illustrative examples of calculating insertion and deletion scores in terms area under curve (AUC), each of which consists of an input (leftmost), a saliency map, and insertion and deletion curves.

**Quantitative Metrics.** Although it is difficult to quantify the fidelity of a saliency map in general, we used two metrics to evaluate the pixel-level relevancy of a given saliency map: deletion and insertion [9]. This quantitative evaluation corresponds to the answer to the first question raised in the beginning of this section. The deletion quantifies the accuracy of finding the smallest susceptive region of an image that is the minimum area to change the model’s prediction when the region is altered. On the other hand, the insertion corresponds to the smallest evidential region that is the part of an image to be preserved to maintain the model prediction. The sole use of deletion is discouraged because, for instance, two extreme cases of the accurate and completely wrong smallest susceptive regions may have an identical deletion score. We therefore used the deletion and insertion metrics to evaluate saliency maps. A higher score results in better insertion, whereas a lower score is preferred for better deletion. In particular, the deletion score prefers a sharp drop when we consider the probability using a function of portion of removed pixels [9], whereas the insertion score is a complementary approach. Figure 5 shows illustrative examples of calculating the metrics.

Both evaluation methods operate by gradually erasing or preserving the input image depending on the importance of pixels in terms of the target class score and measuring the response of the neural network according to the perturbations. This allows us to quantitatively evaluate whether the visual explanation has found an important part of the object that we want to describe in the image. See Algorithms A1 and A2 in the Appendix A for the details on the insertion and deletion metrics, respectively.

**Insertion and Deletion.** We compared the proposed method to three recent methods: RISE [9] and LIME [10], which are representative of the learning-by-perturbation approaches; and Grad-CAM [11], representative of the gradient-based approaches. Besides the settings explained in the previous subsection, we considered another variant of the proposed method by letting $\lambda =10^{-4}$ in Equation (3) to demonstrate the effects of the $\ell_{1}$ regularization, which was the initial value of λ in the hyperparameter search when training the explanation network.

**Speed and Saliency Map Complexity.** In addition, we considered two additional metrics: the speed for generating a saliency map and the complexity of a saliency map. We measured the speed as CPU time taken for a single run of the inference on the target network, which corresponds to the row named Normal inference in Table 3. We excluded LIME in this evaluation due to its excessively long computational time. We measured the complexity of a saliency map as its average pixel intensity, considering that less complexity corresponds to effectively indicating the important region of the input image. For a given dataset X={x}, the pixel intensity is given as $E_{x∼X}\left[∥H_{x}{∥}_{1}\right]$.

#### 4.2.2. Evaluation Results

**Insertion and Deletion.**Table 4 depicts the deletion and the insertion scores of the methods on the four datasets. The results indicate that while RISE performed the best in both scores, our method is comparable to that of RISE. Note that the optimization of λ in the proposed method leads to the non-trivial performance gains of up to 7.5% and 12.3% in the deletion and the insertion scores, respectively. The favorable performance of RISE is probably due to the optimization of a saliency map of an input image at the cost of lengthy computational time. To summarize, the accuracy of the proposed method indicates that it efficiently generates saliency maps of high fidelity as compared to the learning-based methods.

**Speed and Saliency Map Complexity.** We provide the results of the speed and complexity evaluations in Table 3. First, the proposed method runs 814× faster than RISE, and is comparable to Grad-CAM, about 39 fps. This is because the proposed method only requires two individual feed-forward operations on each of the student and the explanation networks, whereas RISE should perform the iterative optimization to create a saliency map as discussed above. Although Grad-CAM is also faster than the proposed method, it lags behind other methods in terms of the fidelity of saliency maps. Moreover, an additional benefit of the proposed method is that the resulting saliency maps are fine-grained, which we quantify as the average pixel intensity of saliency maps in Table 3. The average intensity of the proposed method is only 54% of that of RIME, leading to much sparser saliency maps.

**Discussion.** The better classification accuracy of RISE can be seen in its diffused saliency maps compared to those of our method. This means that the saliency maps from RISE are more likely to cover input features important for the classification better than our method, which we already showed in the experiments in terms of the insertion and deletion scores. Probably, there exists a trade-off between increasing sparsity and classification accuracy when generating saliency maps. While saliency maps of the previous methods faithfully indicate the important input features for the classification, to the best of our knowledge, the analysis that combines the sparsity and the accuracy of saliency maps has hardly been addressed. Additionally, this analysis is worth investigating when we consider a fine-grained classification as a downstream task of a generic classification. The proposed method does not overfit but tends to select most important features so that a small number of features results in a classification accuracy comparable to that of RISE.

### 4.3. Qualitative Evaluations

**Figure 6:**
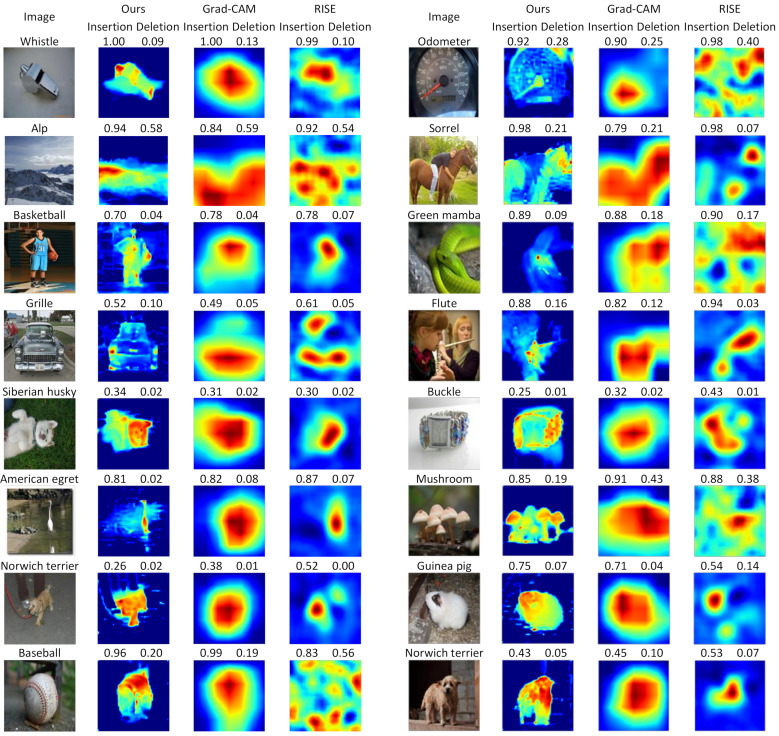
Qualitative results by comparing the saliency maps of the proposed method to the existing methods for the images taken from ImageNet. In the left half of each row, the four columns correspond to input images and the saliency maps of the proposed method, RISE [9], and Grad-CAM [11], respectively, which also applies to the right half of the row. (Best viewed under magnification).

**Figure 7:**
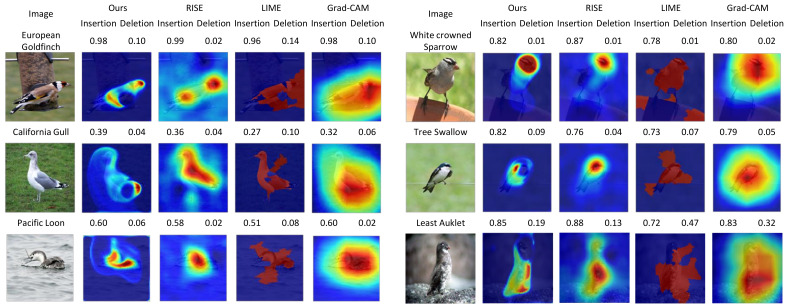
Comparisons of saliency maps from the proposed method to those from RISE [9], LIME [10], and Grad-CAM [11] for *CUB-200* [29]. (Best viewed under magnification).

**Figure 8:**
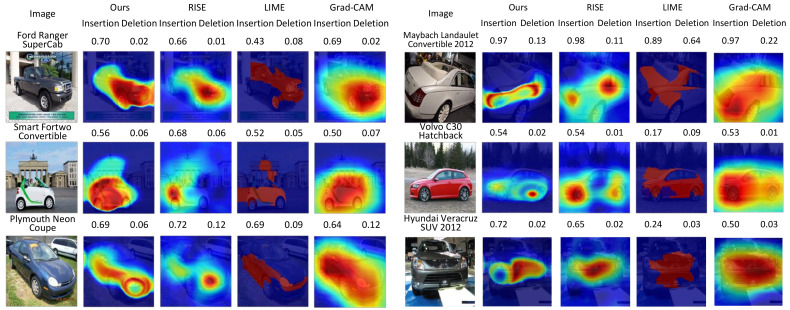
Comparisons of saliency maps from the proposed method to those from RISE [9], LIME [10], and Grad-CAM [11] for *Cars* [30]. (Best viewed under magnification).

**Figure 9:**
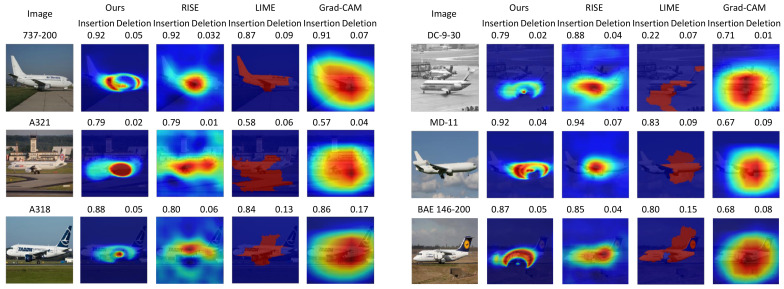
Comparisons of saliency maps from the proposed method to those from RISE [9], LIME [10], and Grad-CAM [11] for *FGVC-Aircraft* [31] variant. (Best viewed under magnification).

We provide a set of visualizations that qualitatively validate the proposed method for the four datasets as shown in Figure 6, Figure 7, Figure 8 and Figure 9. The results demonstrate that the saliency maps that were generated by the proposed method are sparser than those of other methods, and result in faithfully depicting the target object, whereas the results of other methods are blurred and often diffused over the entire region of the image. This capability of representing the target object effectively indicate the core regional clues corresponding to the model prediction. Moreover, the fine-grained characteristics of our saliency maps render them more visually plausible than that of other methods.

In particular, the fine-grained characteristics of the proposed method lead the resulting saliency maps to be similar to segmentation of the target objects, as shown in Figure 6. As RISE depends on the non-deterministic sampling of random masks, its saliency maps are subject to vary and saliency maps may result in excessively distributed blobs. The baseball, odometer, and green mamba are good examples where the saliency maps by the proposed method clearly highlight plausible regions for the target objects. Such a distinction is more obvious in the fine-grained classification. In the case CUB-200 in Figure 7, the proposed method results in much finer saliency maps compared with other methods. The saliency maps of our method indicate the specific clues for the classification, such as the beak for the European goldfinch, the wings for the tree swallow, and the tail for the California gull, respectively. On the other hand, RISE results in coarser saliency maps than those of our method and a much longer computational time. The saliency maps by LIME and Gram-CAM tend to indicate the entire targets. Similar observations were found for the Car and Aircraft datasets, which are shown in Figure 8 and Figure 9, respectively.

**Figure 10:**
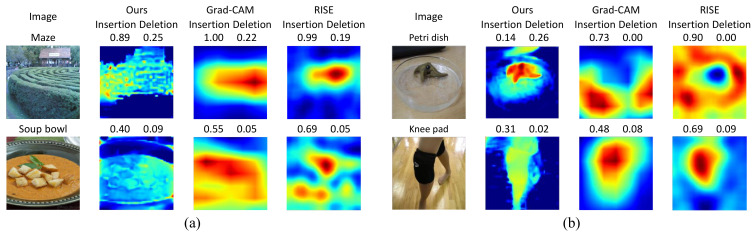
Examples of faulty saliency maps that are generated by the proposed method as compared to the existing methods for the images taken from ImageNet. (**a**) Saliency maps that indicate only some of large regional evidence, and (**b**) saliency maps that focus on faulty regional clues. (Best viewed under magnification)

**Failure cases.**Figure 10 illustrates the failure cases of the proposed method, which were mainly caused by the $\ell_{1}$ regularization of the saliency maps. In particular, these cases usually occurred when the the regional evidence was relatively large and spread over in the input image. In such a case, the regularization led to the proposed method resulting in spotted or dim saliency maps, as shown in Figure 10a. Otherwise, a saliency map may be created in an incorrect location in the image, and thus the corresponding explanation may misclassify the image as different from the original model prediction, as illustrated in Figure 10b.

## 5. Conclusions

We proposed a method to explain the predictions of deep neural networks by learning surrogate networks corresponding to the target network. The surrogate networks in the proposed method consist of two network branches (i.e., the student and the explanation networks). The student network aims to approximate the output of the target network using attention and was trained with the knowledge distillation to better transfer the inference capability of the target network. The explanation network takes the attentive features learned by the student network as inputs. The goal of the explanation network is to generate a saliency map that faithfully retrieves the original class scores of the target network. The experimental results demonstrated the advantages of the proposed method as follows. First, the fidelity of saliency maps generated by the proposed method is competitive in terms of two quantitative metrics (i.e., the deletion and insertion scores) as compared to the best-performing approaches. In addition, the proposed method is efficient in that it runs much faster than the best method by two orders of magnitude. Lastly, the qualitative evaluation indicates that the proposed method results in fine-grained saliency maps and enables itself to be suitable for fine-grained classification, a useful downstream classification task.

## Figures and Tables

**Table 1:** Summary of the datasets used in the experiments. We excluded images that are identified to be in the black list of ImageNet. The aircraft dataset used images of three training and three verification sets as training sets corresponding to the three subcategories.

Dataset	# Classes	Training Set	# Images Validation Set	Test Set
ImageNet [28]	1000	1,281,167	48,238	-
CUB-200 2011 [29]	200	5994	-	5794
Cars [30]	196	8144	-	8041
Aircraft variant [31]	100			
Aircraft family [31]	70	3334	3333	3333
Aircraft manufacturer [31]	30	0	0	0

**Table 2:** Top-1 accuracy of the student network S that is trained with knowledge distillation, compared to T for each of the datasets. For the column ImageNet, we used the non-blacklist images of the ImageNet validation set. We separated the FGVC-Aircraft dataset according to vendor, family, and manufacturer, which are denoted by Aircraft V, Aircraft F, and Aircraft M, respectively.

Dataset	ImageNet	CUB-200	Cars	Aircraft V	Aircraft F	Aircraft M
Target network (T)	0.7615	0.8172	0.8956	0.8402	0.9200	0.9394
Student network (S)	0.7371	0.84	0.8834	0.8483	0.9600	0.9512

**Table 3:** Comparisons of the speed and the pixel intensity of saliency maps of the explanation methods. We measured the speed of processing images taken from ImageNet for each method in frames per second. *Normal inference* represents a single run of the inference on ResNet-50.

	Speed (fps)	Mean Pixel Intensity of a Saliency Map
Normal inference	83.3	1.0
Ours	24.4	0.189
RISE [9]	0.03	0.347
Grad-CAM [11]	34.8	0.421

**Table 4:** Comparisons of the deletion (del) and the insertion (ins) scores of the methods on the datasets. Higher is better for the insertion score, whereas lower is better for the deletion score. Ours ${}_{unopt}$ denotes the unoptimized variant of the proposed method with $\lambda =10^{-4}$. For each dataset, the best and second results are highlighted in red and blue, respectively.

		ImageNet	CUB-200	Cars	Aircraft V	Aircraft F	Aircraft M
**Ours**	ins	0.7049	0.7136	0.7260	0.6910	0.7808	0.8240
	del	0.1211	0.0757	0.0699	0.0746	0.1045	0.1635
**Ours** ${}_{unopt}$	ins	0.6517	0.6895	0.7152	0.6894	0.7726	0.8145
	del	0.1211	0.0659	0.0780	0.0714	0.0978	0.1704
**RISE** [9]	ins	0.7335	0.7461	0.7720	0.7248	0.8026	0.8475
	del	0.1077	0.0588	0.0658	0.0569	0.0762	0.1383
**LIME** [10]	ins	0.6940	0.6531	0.6447	0.5647	0.6532	0.7091
	del	0.1217	0.1287	0.1345	0.1508	0.1935	0.3009
**Grad-CAM** [11]	ins	0.6785	0.6982	0.7197	0.6742	0.7480	0.8011
	del	0.1253	0.0805	0.0798	0.0740	0.1049	0.1735

## Appendix A

This section describes the algorithms used to calculate the deletion and the insertion scores.

**Algorithm A1** Calculating the insertion score.
**Input:** Image *I*, visual explanation *E* of *I*, model *M*, # of pixel batch *n*,
filter size of Gaussian kernel *k*, standard deviation of Gaussian kernel σ
**Output:** Insertion score *s* of *E* for *I*
1: **funtion** INSERTIONSCORE(I,E,M,n,k,σ)
2: c← predicted class of *I* by *M*
3: D←GaussianFilter(I,k,σ)
4: $C\leftarrow \left\{p\left(c|D,M\right)\right\}$
5: i←0
6: **while** I≠D **do**
7: pos← position of *x*th important pixels in E;i<x≤i+n
8: D[pos]←I[pos]
9: $C\leftarrow C\cup \left\{p\left(c|D,M\right)\right\}$
10: i←i+n
11: **end while**
12: s←AUC(C)
13: **end function**

**Algorithm A2** Calculating the deletion score.
**Input:** Image *I*, visual explanation *E* of *I*, model *M*, # of pixel batch *n*
**Output:** Deletion score *s* of *E* for *I*
1: **funtion** DELETIONSCORE(I,E,M,n)
2: c← predicted class of *I* by *M*
3: D←I·0
4: $C\leftarrow \left\{p\left(c|D,M\right)\right\}$
5: i←0
6: **while**I≠D **do**
7: pos← position of *x*th important pixels in E;i<x≤i+n
8: I[pos]←D[pos]
9: $C\leftarrow C\cup \left\{p\left(c|D,M\right)\right\}$
10: i←i+n
11: **end while**
12: s←AUC(C)
13: **end function**
